# Effects of heterologous expression of glycolysis enzymes on product formation in *Clostridium thermocellum*^☆^

**DOI:** 10.1016/j.mec.2026.e00279

**Published:** 2026-05-16

**Authors:** Daniela Prates Chiarelli, Lee R. Lynd, Luana W. Bergamo, Daniel G. Olson

**Affiliations:** a A2G Laboratory, Centro de Biologia Molecular e Engenharia Genética (CBMEG), Universidade Estadual de Campinas (UNICAMP), Campinas, SP, Brazil; b Genetics and Molecular Biology Graduate Program, Instituto de Biologia (IB), Universidade Estadual de Campinas (UNICAMP), Campinas, SP, Brazil; c Thayer School of Engineering, Dartmouth College, Hanover, NH, USA; d Center for Bioenergy Innovation, Oak Ridge National Laboratory, Oak Ridge, TN, USA

**Keywords:** Triosephosphate-isomerase, Fructose-1,6-bisphosphate aldolase, Glyceraldehyde-3-phosphate dehydrogenase, Ethanol, *Clostridium thermocellum*, *Hungateiclostridium thermocellum*, *Ruminiclostridium thermocellum*, *Acetivibrio thermocellus*

## Abstract

Glycolysis is central to *Clostridium thermocellum* metabolism; however, strains engineered for high ethanol titer exhibit a decrease in the conversion of cellobiose to ethanol (i.e., cellobiose-to-ethanol yield), suggesting the presence of glycolytic bottlenecks. We expressed heterologous triosephosphate-isomerases (*tpi)*, fructose-1,6-bisphosphate aldolases (*fba)*, and glyceraldehyde-3-phosphate dehydrogenase (*gapDH)* genes from *Thermoanaerobacterium saccharolyticum* and *Zymomonas mobilis*, along with 26 non-phosphorylating glyceraldehyde-3-phosphate dehydrogenases (*gapN)* variants, to identify limiting reactions. We demonstrated functional expression and increased activity for several Fba and Tpi enzymes in the PPi-free glycolysis engineered strain of *C. thermocellum* (LL1711). Despite *C. thermocellum's* low native FBA activity compared to other industrial strains, increasing Fba or Tpi activity via heterologous expression had no significant effect on cellobiose uptake or ethanol titers in high-substrate fermentations. Furthermore, 25 of 26 tested *gapN* genes had toxic effects on *C. thermocellum* upon transformation. In conclusion, none of the tested glycolytic enzyme modifications improved product titers. These results suggest that the primary metabolic limitation is not at the FBA or TPI reactions, supporting a shift in future engineering efforts toward downstream fermentation pathways.

## Highlights

• We demonstrated functional expression of several heterologous glycolytic enzymes in *Clostridium thermocellum*.
• *C. thermocellum* Aldolase (FBA) has low activity in comparison to other fermentative organisms or thermophiles.
• GAPDH activity is decreased in strains of *C. thermocellum* engineered for increased ethanol production.
• We identified several glycolytic enzymes that are apparently toxic to *C. thermocellum*, mostly *gapN* genes.

## 1 Introduction

Under normal physiological conditions, it has been shown that glycolysis is limited by demand for energy (Boecker et al., 2019; Welch and Scopes, 1985) and metabolic precursors (Lunt and Vander Heiden, 2011), and in some cases, substrate transport (Lagunas, 1993). However, it is not known whether the understanding of glycolysis developed in model organisms such as *Saccharomyces cerevisiae* and *Escherichia coli* is generalizable to non-model organisms such as *Clostridium thermocellum*, which have atypical variants of glycolysis (Zhou et al., 2013). Furthermore, in strains engineered for high-yield and high product titers, glycolysis may become an important limiting factor as it plays a central role in metabolism, degrading glucose into different end-products (Papagianni, 2012; Salvetti et al., 2013).

Prior efforts in some bacteria have targeted the metabolic engineering of different glycolytic pathway steps. In *Corynebacterium glutamicum*, a bacterium used in industry for the production of amino acids, some engineering work in glycolysis has demonstrated that ethanol or alanine titers can be increased by around 1.4-fold and 7.0-fold, respectively, by overexpression of glyceraldehyde-3-phosphate dehydrogenase (GapDH^1^) alone or associated with overexpression of glucose-6-phosphate isomerase (Gpi), 6-phosphofructokinase (Pfk), triosephosphate isomerase (Tpi), and pyruvate kinase (Pyk) (Jojima et al., 2015; Yamamoto et al., 2012).

Engineering of the glycolytic pathway has also been applied as a key strategy to improve product titers in lactic acid bacteria, which are widely used in the food industry and for the production of industrially relevant compounds such as lactic acid and antimicrobial peptides. In *Lactobacillus reuteri,* expression of a truncated version of the *pfk* gene from *Aspergillus niger* was capable of increasing lactate, mannitol and acetate production titers, due to increased enzyme activity and, consequently, glycolytic flux (Papagianni and Legiša, 2014). In *Lactococcus lactis*, a similar strategy was capable of increasing nisin and lactate titers, while enhancing glucose consumption rates (Papagianni, 2012; Papagianni and Avramidis, 2011). In *Lactobacillus plantarum,* overexpression or co-expression of pyruvate carboxylase (Pc), phosphoenolpyruvate carboxykinase (Pepck), and the malic enzyme (Me) enhanced succinic acid production titers in 22-fold, 52.5 mM of succinic acid was produced by the modified strain, in comparison to a concentration of 2.44 mM produced by the wild-type, due to an increase in biomass formation (Tsuji et al., 2013). For *L. plantarum*, expression of pyruvate decarboxylase (Pdc) enzyme, not present in the native organism, could improve ethanol production titers, from 17 mM to 130 mM (Liu et al., 2006). Expression of heterologous fructose-1,6-bisphosphate aldolase (*fba)* gene in *Lactobacillus brevis* could increase both lactic acid production titer (in g/L) and yield (in mol of product/mol of substrate) by around 1.6-fold (Guo et al., 2014).

In *C. thermocellum*, an anaerobic and thermophilic cellulolytic bacterium being considered for the production of ethanol by consolidated bioprocessing (CBP) (Lynd, 2022), engineering of glycolysis has recently gained more attention. This microorganism is capable of solubilizing cellulose at very high rates (2.5 gL^−1^h^−1^) due to its specialized cellulosome structure (Bayer et al., 2004). The highest ethanol titer obtained so far was 30 g/L, achieved by eliminating pathways for competing products (lactate and acetate) followed by adaptive evolution for improved growth (Holwerda et al., 2020). However, in this strain, cellobiose-to-ethanol yield (gram of ethanol/gram of consumed cellobiose) decreased approximately 54% of the theoretical maximum as titer increased. For commercial applications in stand-alone plants, titers of 40-50 g/L ethanol and above 90% of the theoretical maximum yield (considering a theoretical yield of 0.538 g of ethanol/gram of cellobiose) are considered necessary (Lynd, 2022), thus, additional improvements to the ethanol production ability of *C. thermocellum* are needed.

**Fig. 1:**
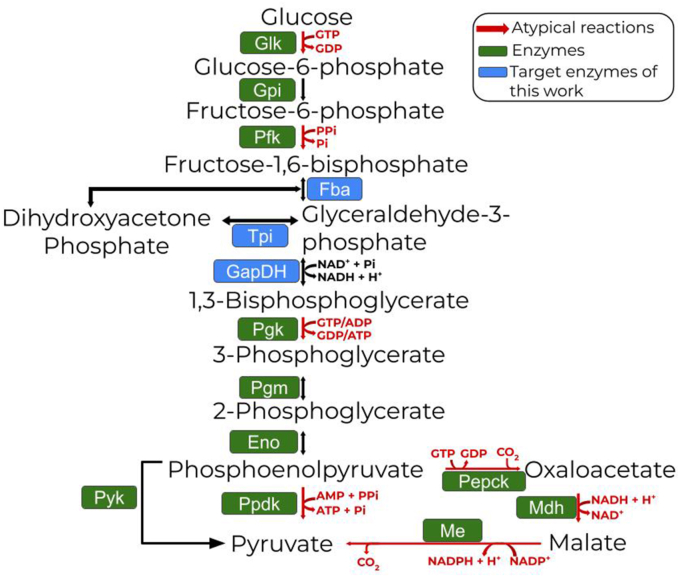
*C. thermocellum* atypical EMP glycolytic pathway. Highlighted in red are the atypical reactions that demand different cofactors than other usual glycolytic pathways. Target reactions of this paper are highlighted in blue squares. Enzymes abbreviations: Glk, glucokinase; Gpi, glucose-6-phosphate isomerase; Pfk, phosphofructokinase; Fba, fructose-1,6-bisphosphate aldolase; Tpi, triosephosphate-isomerase; GapDH, glyceraldehyde-3-phosphate dehydrogenase; Pgk, phosphoglycerate kinase; Pgm, phosphoglycerate mutase; Eno, enolase; Ppdk, pyruvate phosphate dikinase; Mdh, malate dehydrogenase; Me, malic enzyme; Pepck, phosphoenolpyruvate carboxykinase; Pyk, pyruvate kinase.

*C. thermocellum* glycolysis occurs through an atypical Embden-Meyerhof-Parnas (EMP) pathway (Fig. 1) that operates near equilibrium, making it susceptible to product feedback inhibition (Jacobson et al., 2020; Olson et al., 2017; Xiong et al., 2018; Zhou et al., 2013). One approach to overcome this issue might be to alter the organism's atypical reactions. Expression of the *pyk* gene from *Thermoanerobacterium saccharolyticum* (a thermophilic anaerobe that has been engineered to produce ethanol at high yield and titer) in *C. thermocellum,* as a strategy to circumvent some of the atypical reactions of the malate shunt, improved ethanol titers by almost 2-fold, while also increasing the glucose-to-ethanol yield (in mol of ethanol/mol of glucose) (Deng et al., 2013; Olson et al., 2017).

Metabolic flux can also be shifted towards products by replacing *C. thermocellum* PPi-PFK with an ATP-PFK enzyme (Hon et al., 2022). When associated with the deletion of the phosphorylating pyruvate phosphate dikinase (PPi*-ppdk*) gene and expression of the *pyk* gene from *T. saccharolyticum*, ethanol titers were also increased, from 15.1 g/L to 21 g/L (Sharma et al., 2024). Another study focused on the GAPDH reaction of this organism, which has been shown to be the site of a metabolic bottleneck during growth in the presence of added ethanol. Though heterologous expression of *T. saccharolyticum gapDH* in *C. thermocellum* could not increase ethanol production titers, it resulted in increased ethanol tolerance (Tian et al., 2017).

In *C. thermocellum*, allosteric control of several enzymes in central metabolism has been previously demonstrated. The native ATP-linked PFK enzyme is strongly inhibited by PPi (Daley et al., 2023; Kuil et al., 2023), as is its malic enzyme (Taillefer et al., 2015). Malic enzyme is also activated by ammonia (Lamed and Zeikus, 1981; Taillefer et al., 2015). Lactate dehydrogenase is activated by fructose-1,6-bisphosphate (Lo et al., 2015; Özkan et al., 2004; Ponsetto et al., 2025). Based on these prior examples, we hypothesized that additional regulation may be present in glycolysis and that this regulation may be limiting substrate uptake and subsequent product formation.

In this work, we set out to identify reactions in glycolysis that may be limiting product titer. Our approach was to express heterologous glycolytic enzymes from various other thermophilic organisms as a strategy to circumvent limitations to product titer from native enzymes (e.g., from allosteric regulation). Since we have previously studied the effects of heterologous expression of ATP-Pfk in *C. thermocellum* (Hon et al., 2022), we decided to focus on the enzymes immediately downstream: Fba, Tpi, and GapDH. In addition, we tested some non-phosphorylating GapN enzymes, a variant of GapDH that avoids substrate-level phosphorylation (and its associated energy conservation), therefore leading to a higher thermodynamic driving force, which may be important for increasing product titer if titer is constrained by pathway thermodynamics (Dash et al., 2019; Noor et al., 2014; Valverde et al., 1999). Since we were building off the work of Hon et al., we also decided to perform our genetic manipulations in strain LL1711, a strain developed in that work, where the native PPi-linked PFK has been replaced with an ATP-linked PFK.

## 2 Methods

### 2.1 Strains and plasmids

Genes from Table 1 were synthesized and cloned into the pDGO143 plasmid backbone and driven by the P2638 promoter, (Accession Number: KX259110.1) (Hon et al., 2016) by the U.S. Department of Energy Joint Genome Institute (JGI). Plasmids were transformed into T7 Express Competent *E. coli* cells (NEB C2566). *Dcm* methylation is not present in this strain, which is important for later transformation into *C. thermocellum*, increasing transformation efficiency (Guss et al., 2012).

**Table 1:** Genes expressed in *C. thermocellum* in this work. The corresponding protein, the genome origin of each selected gene, the genome NCBI accession number and the gene location in the genome (Locus Tag) are listed.

Protein	Organism	NCBI Accession Number	Locus Tag
Triosephosphate isomerase EC 5.3.1.1	*Thermoanaerobacterium saccharolyticum*	CP003184	tsac_2312
			tsac_2484
	*Zymomonas mobilis*	AE008692	zmo_0465
Fructose-bisphosphate aldolase, class II EC 4.1.2.13	*Thermoanaerobacterium saccharolyticum*	CP003184	tsac_2313
			tsac_0260
			tsac_0328
	*Zymomonas mobilis*	AE008692	zmo_0179
Glyceraldehyde 3-phosphate dehydrogenase EC 1.2.1.12	*Thermoanaerobacterium saccharolyticum*	CP003184	tsac_2486
Nonphosphorylating glyceraldehyde-3-phosphate dehydrogenase EC 1.2.1.9	*Acidilobus saccharovorans*	CP001742	asac_1423
	*Aeropyrum camini SY1, JCM 12091*	AP012489	acam_0129
	*Halalkalibacterium halodurans*	CP040441	bh_2237
	*Bacillus methanolicus MGA3*	CP007739	ga0248213_103193
	*Clostridium acetobutylicum*	AE001437	cac_3657
	*Clostridium tepidiprofundi SG 508*	NZ_LTBA00000000	clte_13480
	*Clostridium thermobutyricum DSM 4928*	NZ_LTAY00000000	ga0336823_070_97578_99023
	*Desulfacinum hydrothermale DSM 13146*	GCF_900176285.1	ej40draft_01996
	*Desulfacinum infernum DSM 9756*	GCF_900129305.1	ej47draft_02370
	*Desulfurobacterium thermolithotrophum BSA, DSM 11699*	CP002543	dester_0320
	*Desulfurococcus fermentans Z-1312, DSM 16532*	CP003321.1	desfe_0067
	*Pseudothermotoga hypogea DSM 11164*	NZ_CP007141.1	bs13draft_1691
	*Pyrococcus furiosus*	NZ_CP023154	pf_0755
	*Rubrobacter xylanophilus DSM 9941*	CP000386.1	rxyl_0759
	*Saccharolobus solfataricus 98/2*	ACUK00000000.1	ssol98_010100011170
	*Staphylothermus hellenicus P8, DSM 12710*	CP002051.1	shell_0726
	*Streptococcus infantarius infantarius CJ18*	CP003295.1	sinf_0586
	*Streptococcus mutans*	AE014133.2	smu_676
	*Sulfodiicoccus acidiphilus HS-1*	AP018553.1	ga0309577_112362
	*Sulfolobus islandicus L.D.8.5*	NC_013769.1	ld85_2431
	*Sulfurihydrogenibium sp. YO3AOP1*	CP001080.1	syo3aop1_1075
	*Thermococcus kodakarensis*	AP006878.1	tk_0705
	*Thermoproteus tenax*	NC_016070	ttx_1169
	*Unclassified "LHC4-2-B″ archaeon JGI MDM2 LHC4sed-1-M18 (unscreened)*	2643221513^a^	ga0097925_10921
	*Unclassified bacterium JGI MDM2 M14Mat9-1-D14 (unscreened)*	2734482262^a^	ga0192188_1141
	*Unclassified Euryarchaeota archaeon JGI MDM2 SSWsed-3-L11 (unscreened)*	2645728132^a^	ga0097871_11911

a Organisms unscreened and unclassified that do not have an NCBI accession number. Numbers shown refer to genome ID from JGI's IMG database.

A strain of *C. thermocellum* engineered for a PPi-free glycolysis, strain LL1711 (Accession number: SRX9409011) (Sharma et al., 2024), and the wild-type strain, LL1004 (Accession number: CP002416), were used for fermentation assays and enzyme assays. The parental strain of LL1711, strain LL1592 (Accession number: SRX5290154) (Hon et al., 2022), was also used for GAPDH activity analysis to identify the point in its lineage at which the enzyme activity changed.

CTFUD (Olson and Lynd, 2012) rich medium was used for genetic manipulations, and MTC-5 chemically defined medium (Hon et al., 2017), supplemented with 30 g/L of cellobiose, was used for fermentations and the preparation of cell pellets for enzyme assays.

### 2.2 Plasmids extraction

*E. coli* strains carrying the plasmids with genes from Table 1 were grown in 10 mL LB media with 100 μg/mL of carbenicillin at 37 °C overnight. Plasmids were extracted with the Monarch® Plasmid Miniprep Kit (New England Biolabs-NEB) or Fast-n-Easy Plasmid Miniprep Kit (Cellco Biotech do Brasil Ltda) and stored at −20 °C for further use in *C. thermocellum* transformation and sequencing for confirmation of constructs.

### 2.3 Bacterial transformation

*C. thermocellum* transformation was performed as described by Olson and Lynd (2012). After growth in CTFUD medium at 55 °C, cells were harvested at an optical density (OD_600_) of 0.6. After three ultrapure water washes and centrifuging, 25 μL of cell suspension was used for transformation with 0.5 to 1.0 μg of DNA. After transformation, cells were grown overnight at 50 °C in CTFUD-rich medium. Overnight cultures were plated by pouring cell cultures into CTFUD agar medium containing 6 μg/mL thiamphenicol and incubated at 55 °C for 3 to 7 days. All described steps, except centrifugation, were performed under anaerobic conditions in a Coy anaerobic chamber with a gas phase of 85% N_2_, 10% CO_2_, and 5% H_2_ (Coy Laboratory Products, Grass Lakes, MI, USA) (Olson and Lynd, 2012).

### 2.4 Protein expression by an inducible system

pLZ2 plasmid (Zhang et al., 2018) carrying *Streptococcus mutans gapN* gene under inducible arabinose promoter was transformed into *E. coli* BL21(DE3) using standard electroporation protocol. Gene expression was induced by inoculating a transformed colony in 20 mL of LB supplemented with 2 g/L of arabinose and growing at 37 °C, 200 rpm for 24 h. After this time, pellets were harvested by centrifugation and stored at −80 °C for further use as a control in enzymatic assays.

### 2.5 Fermentations and product quantification

Fermentation profiles of transformants were evaluated by collecting *C. thermocellum* colonies from transformation plates and inoculating them in 2 mL tubes containing 1.5 mL of MTC-5 medium supplemented with 30 g/L cellobiose and 6 μg/mL thiamphenicol. They were incubated statically at 55 °C in the same medium for 4 days, under anaerobic conditions in a Coy anaerobic chamber with a gas phase of 85% N_2_, 10% CO_2_, and 5% H_2_ (Coy Laboratory Products, Grass Lakes, MI, USA). Strains carrying the pDGO143 plasmid were used as an empty-vector control.

The fermentation products were quantified using a Waters™ HPLC (Milford, MA) system, equipped with a refractive index and UV detector, and an Aminex HPX-87H column (Bio-Rad, Hercules, CA) as previously described (Holwerda et al., 2014).

### 2.6 Whole genome sequencing

Strains LL1711, LL1592, and LL1004 carrying the *T. saccharolyticum gapDH* expression plasmid were grown in MTC-5 chemically defined medium (Hon et al., 2017) until reaching an OD_600_ of 1.0. Then they were harvested, and pellets were washed with a 1X Phosphate-buffered saline solution (137 mM NaCl, 2.7 mM KCl, 10 mM Na_2_HPO_4,_ and 1.8 mM KH_2_PO_4_) at a pH of 7.4. Pellets were then resuspended in 1X DNA/RNA Shield™ Ready-to-use (Zymo Research, R1100-250) and sent for sequencing by a commercial provider. Bacterial genome sequencing was then performed by Plasmidsaurus using Oxford Nanopore Technology with custom analysis and annotation. Annotation was performed with Bakta v1.11, contig analysis was done by Bandage v0.8.1, genome contamination was checked with CheckM v1.2.2, and for species identification, both Mash v2.3 and Sourmash v4.6.1 analysers were utilized.

### 2.7 Enzyme assays

Different colonies from the same transformation were inoculated in 10 mL of MTC-5 medium supplemented with 30 g/L cellobiose and 6 μg/mL thiamphenicol and grown until reaching an OD_600_ of 0.4 - 0.6. After growth, cultures were centrifuged, washed with 100 mM Tris-HCl buffer at the same pH of the assay (as described in the following topics), and centrifuged again. Pellets were lysed with 0.1 mg of lysozyme for 10 min at room temperature, followed by the addition of DNase I (to reduce viscosity associated with DNA) and incubation for 10 min. Lysed cells were centrifuged, and the supernatant (cell-free extracts - CFE) was used for enzymatic assays as described below.

Assays were performed aerobically in a Cary 3500 Multicell UV-Vis Spectrophotometer, using 1 cm quartz cuvettes, at 55 °C. For GapN enzymes, assays were performed at both 55 °C and 30 °C. All reactions were started by the addition of the substrate.

#### 2.7.1 Triosephosphate-isomerase (TPI - EC 5.3.1.1)

The TPI assay was performed based on NADH oxidation using a coupling enzyme strategy, according to Patni and Alexander's (1971) protocol, with modifications. The reaction mixture consisted of 50 mM Tris-HCl buffer at pH 7.5, 0.3 mM NADH, 1 mM glyceraldehyde-3-phosphate as substrate, 4 units (U) of α-glycerophosphate-dehydrogenase (Sigma-Aldrich, G1881) as coupling enzyme, and 1 to 2 μL of cell-free extract (CFE) dilutions, to a total volume of 100 μL. NADH consumption over time was monitored at 340 nm (*ε* = 6,2 mM^−1^cm^−1^). TPI enzyme 5.9 mg/mL (Sigma-Aldrich, T2391) at 4U was used as a control for assay validation (Patni and Alexander, 1971).

#### 2.7.2 Fructose-1,6-bisphosphate aldolase (FBA - EC 4.1.2.13)

The FBA assay was adapted from the TPI protocol described above. The reaction mixture consisted of 50 mM Tris-HCl buffer at pH 7.5, 0.3 mM NADH, 1 mM fructose-1,6-bisphosphate as substrate, 4 U α-glycerophosphate-dehydrogenase (Sigma-Aldrich, G1881) as coupling enzyme, 4 U Tpi enzyme (Sigma-Aldrich, T2391), and 10 to 15 μL of cell-free extract (CFE). NADH consumption over time was measured at 340 nm (*ε* = 6,2 mM^−1^cm^−1^). FBA enzyme (Sigma-Aldrich, A8811) at 4 U was used as a control for assay validation (Patni and Alexander, 1971)**.**

#### 2.7.3 Glyceraldehyde-3-phosphate dehydrogenase (GAPDH - EC 1.2.1.12)

The GAPDH assay was performed as described by Tian et al. (2017) and Byers et al. (1979). Assay contained 50 mM Tris-HCl buffer at pH 7.5, 10 mM sodium arsenate, 10 mM glyceraldehyde-3-phosphate as substrate, 0.5 mM NAD^+^, and 10 to 15 μL of cell-free extract (CFE), to a total volume of 100 μL. NADH formation over time was read at 340 nm (*ε* = 6,2 mM^−1^cm^−1^)(Byers et al., 1979; Tian et al., 2017). GapDH enzyme (Sigma Aldrich, G2267) at 4U was used as a control for assay validation.

#### 2.7.4 Non-phosphorylating glyceraldehyde-3-phosphate dehydrogenase (GAPN - EC 1.2.1.9)

The GAPN assay was performed as described by Mateos and Serrano (1992). Assay contained 50 mM Tris-HCl buffer at pH 8.5, 1 mM glyceraldehyde-3-phosphate as substrate, 0.5 mM NAD(P)^+^, and 10 to 15 μL of cell-free extract (CFE), to a total volume of 100 μL. NAD(P)H formation over time was read at 340 nm (*ε* = 6,2 mM^−1^cm^−1^)(Mateos and Serrano, 1992). Cell-free extract of *E. coli* expressing *S. mutans gapN* gene (pLZ2 plasmid)(Zhang et al., 2018) was used as a control.

### 2.8 Statistical analysis

Statistical analyses were performed in Python, using the SciPy library. Normality and homoscedasticity were evaluated via Shapiro-Wilk and Levene's tests, respectively. Given the presence of heteroscedasticity (p < 0.05), a two-tailed unpaired Welch's *t*-test was employed to compare group means, as it accounts for unequal variances by adjusting the degrees of freedom. The significance level was set at α = 0.05. Due to the limited sample size (n < 3), normality was assumed for samples tsac_2313 and zmo_0465 to proceed with Welch's *t*-test analysis.

## 3 Results and discussion

### 3.1 Gene selection

*T. saccharolyticum* and *Z. mobilis* are known for their capacity to produce and tolerate high ethanol titers (Doelle and Greenfield, 1985; Jacobson et al., 2020; Rogers et al., 2007). As previous studies have demonstrated, it is possible to successfully express *T. saccharolyticum* genes in *C. thermocellum* (Chiarelli et al., 2024; Hon et al., 2017, 2022; Huang et al., 2025; Mazzoli and Olson, 2020; Sharma et al., 2024; Tian et al., 2017). Although *Z. mobilis* is a mesophilic organism with an optimum growth temperature around 30 °C, its glycolytic enzymes have been shown to allow conversion of glucose to ethanol at temperatures up to 50 °C (Scopes and Griffiths-Smith, 1986), and several of its alcohol dehydrogenases have been successfully expressed in *C. thermocellum* (Chiarelli et al., 2024). Therefore, all genes annotated as aldolases, triosephosphate isomerases, or glyceraldehyde 3-phosphate dehydrogenase from these two organisms were selected for expression in *C. thermocellum.*

Since *gapN* is not present in either *T. saccharolyticum* or *Z. mobilis*, we pursued a different approach for this enzyme. There are two distinct types of GapN enzyme: bacterial (Centeno-Leija et al., 2013) and archaeal (Ito et al., 2014). In the KEGG Orthologous Groups database (Kanehisa et al., 2016), K00131 is associated with the bacterial type of GapN enzyme, and K18978 is associated with the archaeal type of GapN enzyme. To identify putative thermophilic GapN enzymes, we searched the JGI IMG database (https://img.jgi.doe.gov/) (Markowitz et al., 2012) for genomes annotated with Organism Temperature Range values classified as “Thermophile” or “Hyperthermophile,” resulting in ∼870 genomes. Within those genomes, we searched for genes associated with the K00131 or K18978 KO IDs. This resulted in a set of 26 *gapN* genes, which were all used in the present work.

### 3.2 GAPN and GAPDH assay validation

After transforming *C. thermocellum* with several *gapN* plasmids from Table 1, enzyme assays were performed to check for enzyme expression and activity. *C. thermocellum* naturally possesses the GAPDH reaction, but not GAPN. However, since both reactions are very similar, we wanted to confirm that we could distinguish them. To do this, we performed both assays on *E. coli* BL21 cell extracts with no plasmid (i.e., where only GAPDH activity from the native *gapDH* gene is present), as well as *E. coli* BL21 cells with the pLZ2 plasmid expressing *S. mutans gapN*.

NAD^+^-linked GAPN enzyme activity might be detected when performing GAPDH assays; however, the opposite does not occur (Mateos and Serrano, 1992). Although most GapN enzymes have NADP^+^-linked activity, two have been described as NAD^+^-linked: the ones from *Thermoproteus tenax* and *Thermococcus kodakarensis* (Brunner et al., 1998; Mallinson et al., 2023; Matsubara et al., 2011). To differentiate activities, *C. thermocellum* or *E. coli* BL21 strains without *gapN* genes were used as negative controls for all GAPN assays. Table 2 illustrates the differences between assays, using as an example activities obtained for BL21 and BL21 carrying the *S. mutans gapN* gene.

**Table 2:** Specific activity for *E. coli* BL21 strains with or without plasmid pLZ2, expressing the *S. mutans gapN* gene.

Heterologous Gene	Assay	Cofactor	Specific Activity (μmol/min/mg CFE^a^)
None	GAPN	NADP+	0
None	GAPN	NAD+	0
None	GAPDH	NADP+	0
None	GAPDH	NAD+	0.31
smu_676	GAPN	NADP+	2.49
smu_676	GAPN	NAD+	0
smu_676	GAPDH	NADP+	2.74
smu_676	GAPDH	NAD+	0.1

a CFE = Cell-free extract.

### 3.3 Expression and enzyme activity assessment in *C. thermocellum*

When working with heterologous expression, transformation efficiency can give the first insights into how exogenous genes affect host cell metabolism. Low transformation efficiency relative to the empty-vector control can be caused by either DNA-related effects (e.g. introduction of targets for restriction enzymes or CRISPR systems), or protein function effects (e.g. high gene or protein expression can cause a metabolic burden in the bacterium, compete with native proteins for substrates, or induce mutations in essential genes, leading to death or growth deficiency of the host cell) (Rosano and Ceccarelli, 2014; Saida et al., 2006).

All *fba*, *tpi,* and *gapDH* genes from Table 1 were successfully transformed into *C. thermocellum* PPi-free glycolysis strain LL1711. Transformation efficiencies were similar to those obtained for the empty vector control, ranging from 10^4^ to 10^5^ CFU/μg of DNA, with lower efficiency for the transformations with *zmo_0179*, *tsac_2312*, and *tsac_0260* genes (10^3^ CFU/μg of DNA).

For *gapN* genes, functional expression could decrease energy conservation (i.e., production of ATP/GTP in glycolysis) and alter redox balance (i.e., producing NADPH instead of NADH in glycolysis). Either of these effects could inhibit cell growth. Since expression of the GAPN enzyme could disturb ATP metabolism by competing for G3P substrate, thereby skipping the ATP-producing Pgk reaction (Komati Reddy et al., 2015; Takeno et al., 2010; Valverde et al., 1999), we expected that the transformation of *C. thermocellum* with this gene would be more challenging.

**Fig. 2:**
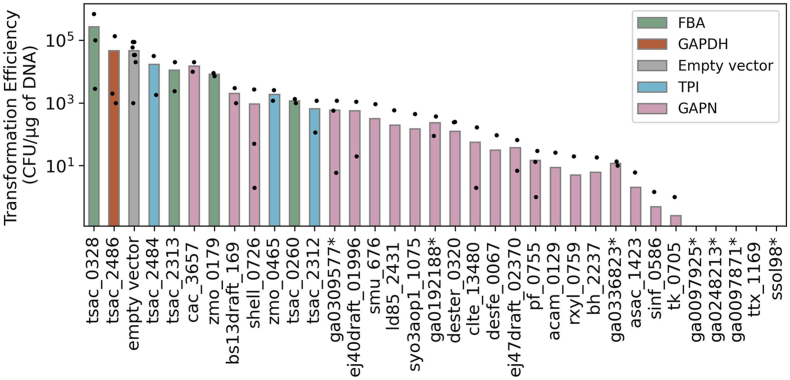
Transformation efficiency of plasmids from Table 1 transformed into the PPi-free glycolysis strain of *C. thermocellum*, LL1711. Plasmid pDGO143 was used as an empty vector control. Results are shown on a logarithmic scale. Dots represent experimental replicates. ∗Locus tag IDs were truncated for display purposes; check Table 1 for complete IDs.

From the 26 plasmids carrying *gapN* genes (Table 1), 21 plasmids could be transformed. Except for the plasmid carrying the *cac_3657* gene, which showed a transformation efficiency of 10^4^ CFU/μg of DNA, the other 20 exhibited transformation efficiencies 10-10,000 fold lower than the empty-vector control. We included *gapN* from *C. acetobutylicum* (*cac_3657*), as it is one of the better-characterized bacterial GapN enzymes. However, since *C. acetobutylicum* is a mesophilic organism, the high transformation efficiency (likely due to a lack of function at thermophilic temperatures) was not surprising. The transformation of the remaining 5 plasmids harboring *gapN* genes resulted in no colonies even after two or three trials (Fig. 2).

Although we did not observe any GAPN activity in any of our transformants (data not shown), the reduced transformation efficiency provides some limited (albeit negative) evidence that some of the tested *gapN* genes may be metabolically active in *C. thermocellum*, possibly causing a metabolic burden in the host due to protein expression or incorrect folding (Baneyx and Mujacic, 2004; Glick, 1995).

Observation of increased enzyme activity provides strong evidence for functional expression of candidate enzymes. Although we primarily focused on gene expression in the LL1711 strain of *C. thermocellum* that had been previously engineered for increased ethanol production by expression of an ATP-linked Pfk enzyme (Hon et al., 2022), we also included the WT strain of *C. thermocellum* (LL1004) as a control to determine whether any of our previous engineering work had altered enzyme activities for the FBA, TPI, and/or GAPDH reactions.

**Fig. 3:**
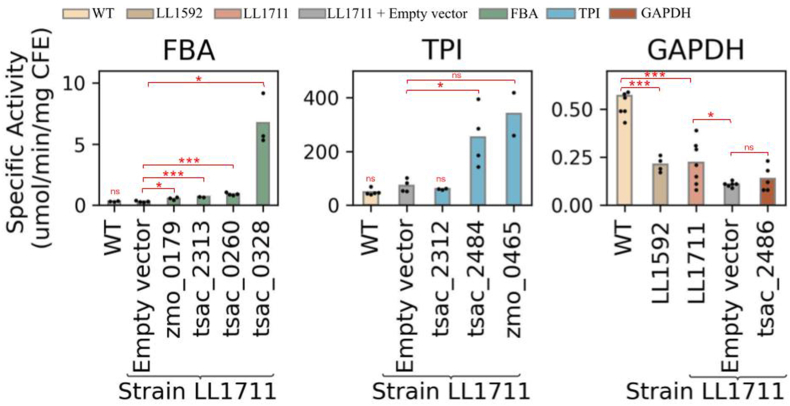
Specific activity for FBA (A), TPI (B), and GAPDH (C) for the PPi-free glycolysis strain (LL1711), the wild-type strain (LL1004), the parental strain of LL1711 (LL1592), and the LL1711 strain expressing heterologous genes. Dots represent different colonies from the same transformation (i.e., independent biological replicates). Asterisks in red represent the significance level of the Welch's *t-*test. One asterisk indicates p-value <0.05, two asterisks indicate p-value <0.01, three asterisks indicate p-value <0.001, and ns indicates no significance. Bars in green refer to strain LL1711 carrying a *fba* gene, bars in blue refer to strain LL1711 carrying a *tpi* gene, bars in brown refer to strain LL1711 carrying a *gapDH* gene, and bars in gray represent LL1711 carrying the empty vector control plasmid.CFE = cell-free extract.

For the FBA reaction, all tested enzymes exhibited significant activity above the empty vector control, with the largest increase in activity observed for *tsac_0328* (Fig. 3). Interestingly, WT levels of FBA are relatively low compared to the observed levels of cellobiose uptake in strain LL1711. Based on batch fermentation data of strain LL1711 (Sharma et al., 2024), performed in a 400 mL working volume with 100 g/L of cellobiose and pH controlled at 7.0, and using a conversion of 0.47 gDCW/OD_600_ (Sharma et al., 2023), we can estimate a cellobiose uptake rate of 6 mM cellobiose/hr/gDCW. Assuming that cells are composed of approximately 50% protein, this implies an FBA flux of 0.395 μmol/min/mg CFE, slightly higher than our measured value of 0.30 ± 0.05 μmol/min/mg. Furthermore, the measured values for *C. thermocellum* aldolase activities are lower compared to other bacteria, such as *E. coli* (4.6 μmol/min/mg), *Thermus aquaticus (*1.6 μmol/min/mg), and *C. glutamicum* (0.38 – 0.55 μmol/min/mg), *Lactobacillus rhamnosus* (3.8 ± 0.16) and *Bacillus stearothermophilus* (0.4), though slightly higher than in *Thermoanaerobium brockii* (0.23) (Table 3), all bacteria of industrial relevance that follow a conventional EMP-pathway (Baldwin et al., 1978; Guo et al., 2014; Hashizume et al., 1976; Jojima et al., 2015; Lamed and Zeikus, 1980; Montigny and Sygusch, 1996; Yamamoto et al., 2012).

**Table 3:** Comparison of literature data for aldolase specific activities (in μmol/min/mg) reported for cell-free extract of different bacteria and aldolase activity in *C. thermocellum* cell-free extract observed in this work.

Organism	Aldolase Specific Activity (μmol/min/mg CFE)	Reference
*Escherichia coli*	4.6	Baldwin et al. (1978)
*Lactobacillus rhamnosus*	3.8	Guo et al. (2014)
*Thermus aquaticus*	1.6	Montigny and Sygusch (1996)
*Corynebacterium glutamicum*	0.38 – 0.55	Jojima et al. (2015); Yamamoto et al., 2012
*Bacillus stearothermophilus*	0.4	Hashizume et al. (1976)
*Clostridium thermocellum* ATCC 27405	0.95	Hogsett (1995)
*Clostridium thermocellum* DSM 1313	0.30	This work
*Thermoanaerobium brockii*	0.23	Lamed and Zeikus (1980)

**Fig. 4:**
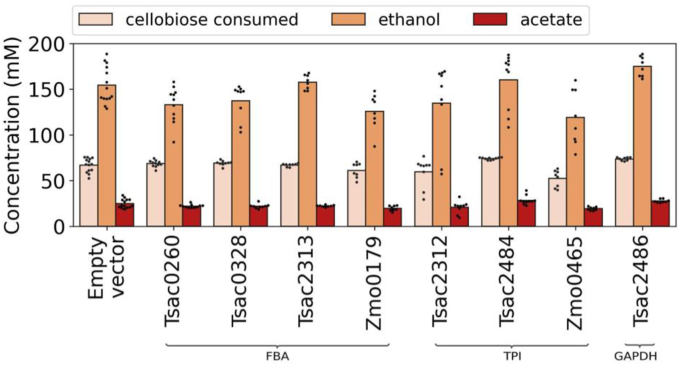
Cellobiose consumed (in mM), acetate and ethanol titers (in mM) observed for the PPi-free glycolysis strain (LL1711) expressing either an *fba*, *tpi,* or *gapDH* gene after 96h of fermentation in MTC-5 media with ∼30 g/L cellobiose. Dots represent different colonies from the same transformation (i.e., independent biological replicates). Strain LL1711 carrying the pDGO143 plasmid was used as an empty vector control. Student's *t-*test showed significance in comparison to the empty vector for Zmo0179 and Zmo0465 (p-value <0.05) and was not significant for other genes tested (p-value >0.05).

Despite this circumstantial evidence suggesting that FBA might limit glycolytic flux, increasing FBA activity through the heterologous expression of various Fba enzymes had no effect on substrate consumption or product formation (Fig. 4). One possible explanation is that we are underestimating the true levels of FBA activity (for example, by omitting a necessary activator). Another possible explanation is that we are overestimating glycolytic flux due to the compilation of data from heterogeneous data sets. Due to the lack of effect of heterologous Fba expression on fermentation phenotypes, we did not investigate this further.

For the TPI reaction, two of three heterologous enzymes showed significantly increased activity compared to the empty vector control (tsac_2484 and zmo_0465) (Fig. 3). However, the WT levels of TPI activity were relatively high (50 μmol/min/mg) relative to the observed flux (0.395 μmol/min/mg calculated in the previous paragraph). As expected, increasing TPI activity by heterologous expression did not affect fermentation products (Fig. 4).

For the GAPDH reaction, we also observed lower than expected activity in our empty vector control strain (0.15 μmol/min/mg CFE) relative to the expected value (0.790 μmol/min/mg CFE). Note that this is two times higher than the predicted value for FBA and TPI due to stoichiometric effects. To better understand the reason for this low activity, we measured activity in several ancestor strains: LL1711, LL1592, and WT. We observed a significant decrease in activity between the WT and LL1592 strain, and then another smaller decrease when we introduced the empty vector plasmid (Fig. 3).

We hypothesized that some mutation might have occurred during the manipulation of these strains, leading to decreased activity. However, re-sequencing of LL1711, with and without empty vector, and LL1592 strains did not show mutations in this gene, in comparison to the wild-type strain (Accession Number: PRJNA1371017) (Supplementary Table S1). To test whether the GAPDH reaction was limiting glycolysis, we attempted to express a GapDH enzyme from *T. saccharolyticum* (Tsac_2486). Previously, we have shown that this enzyme increases activity in WT *C. thermocellum* (Tian et al., 2017); however, we did not observe a similar effect in strain LL1711 (Fig. 3).

Based on previous observations from Hon et al. (2022), when we initiated these experiments, we suspected that the GAPDH reaction might be limiting glycolysis in strains with ATP-linked Pfk enzymes, due to unchanged levels of dihydroxyacetone phosphate (DHAP) (Hon et al., 2022). Subsequent work from Sharma et al. (2024) with this strain (LL1711) demonstrated that there is, in fact, a large increase in DHAP and several downstream metabolites during growth phase of fermentation; however, this increase is only transient, as abundance of this metabolite was again reduced after completion of fermentation (Sharma et al., 2024), and was likely not observed in our previous work due to the measurement of intracellular metabolites at only a single time point. Thus, although the heterologous GapDH expression experiment in the current work was inconclusive, we decided not to pursue it further in light of other experimental evidence (Sharma et al., 2024). Future work might focus on analysing a possible reduction in *gapdh* gene expression.

### 3.4 Evaluation of fermentation profiles

After demonstrating successful expression of several glycolytic genes in *C. thermocellum*, we were interested in observing any changes in fermentation profiles (i.e., substrate consumption or product formation). We performed fermentations with high concentrations (30 g/L) of cellobiose. At this substrate concentration, fermentation stops before the substrate has been completely consumed.

We hypothesized that increased activity of glycolytic enzymes might allow continued substrate consumption as observed in previous works in the literature (Emmerling et al., 2000; Papagianni, 2012); however, we did not observe any such changes. For all of the enzymes tested (4 Fba candidates, 3 Tpi candidates, and 1 GapDH candidate), there was no significant effect on cellobiose consumption or product formation (ethanol or acetate production) (Fig. 4). Additional fermentation products were measured, including lactate, pyruvate, glucose, formate, lactate, malate and succinate; however, no changes were observed in those metabolites either (Supplementary Table 1).

Previous works report promising results in improving glycolytic flux while overexpressing mid-glycolysis enzymes such as GAPDH and FBA in *L. brevis, C. glutamicum,* and *C. thermocellum* (Guo et al., 2014; Tian et al., 2017; Yamamoto et al., 2012), all performed with wild-type strains. Here, we worked with an already engineered strain, suggesting that heterologous expression in wild-type and engineered bacteria might work differently. Co-expression of these enzymes has also been suggested as a strategy to achieve better results and could be a subject for future work in engineered strains of *C. thermocellum* (Jojima et al., 2015).

Previous works have also shown that *C. thermocellum* has a highly thermodynamically constrained glycolytic pathway in comparison to other organisms such as *E. coli* and *Z. mobilis*. This way, overexpression of individual glycolytic enzymes might not cause any effects due to this organism's EMP pathway already working with increased enzyme burden and being more susceptible to allosteric regulation (Khana et al., 2025; She et al., 2025).

As expression of *gapN* genes showed no GAPN enzyme activity in *C. thermocellum* (data not shown), fermentation assays were not performed with these strains. Despite these negative results, the low transformation efficiency of these genes suggests that some of them may be functional, providing useful information for future work expressing *gapN* genes in this organism, particularly if better inducible promoter systems can be developed to allow conditional expression of genes that would otherwise be toxic.

## 4 Conclusions

We demonstrated functional expression of several exogenous glycolytic enzymes in *C. thermocellum*, including three Fba enzymes and two Tpi enzymes. We observed that WT *C. thermocellum* has relatively low levels of FBA activity; however, we were able to rule out this factor as a limitation to sugar uptake in this organism by successfully expressing heterologous Fba enzymes. Similarly, we also ruled out low TPI activity as a factor limiting sugar uptake. Surprisingly, we observed that some of our previous strain engineering efforts appear to have decreased GAPDH activity, particularly the introduction of the ethanol production pathway from *T. saccharolyticum* (LL1004 → LL1592) (Chiarelli et al., 2024; Hon et al., 2018). Although we did not find obvious genetic evidence that can explain the decreased activity, it may be an interesting area for future investigation.

We also observed some indications that non-phosphorylating glyceraldehyde-3-phosphate dehydrogenase (GAPN) enzymes could be toxic to *C. thermocellum*, as evidenced by the low transformation efficiencies associated with the lack of enzyme activity, though further investigation is still needed. Previously, we have observed that high titers of ethanol cause a local thermodynamic equilibrium at the GAPDH reaction (Tian et al., 2017), and this appears to be associated with the usage of the same cofactor pair (NADH/NAD^+^) by the GAPDH and alcohol dehydrogenase reactions (Pech-Canul et al., 2024; Tian et al., 2019). The GAPN reaction may enable us to overcome this thermodynamic bottleneck, since it provides increased thermodynamic driving force at the expense of energy conservation.

When the GAPDH reaction is replaced by the GAPN reaction, glycolysis becomes energy-neutral. However, since most GapN enzymes use NADPH instead of the NADH cofactor used by the *C. thermocellum* GapDH reaction, in addition to the thermodynamic equilibrium effect, there is also a change in redox balance. Finding ways to distinguish between these effects, such as engineering the cofactor specificity of GapN enzymes (Mallinson et al., 2023) or introducing ethanol production pathways that are compatible with NADPH-linked GapN enzymes (Dash et al., 2019), may offer potential solutions to this problem. Other potential ways to better characterize the function of GapN enzymes in *C. thermocellum* include the development of tightly controlled inducible promoters and/or the identification of growth conditions where glycolytic genes are not essential.

## CRediT authorship contribution statement

**Daniela Prates Chiarelli:** Writing – review & editing, Writing – original draft, Visualization, Methodology, Formal analysis, Data curation, Conceptualization. **Lee R. Lynd:** Writing – review & editing, Supervision, Funding acquisition. **Luana W. Bergamo:** Writing – review & editing, Supervision. **Daniel G. Olson:** Writing – review & editing, Writing – original draft, Visualization, Supervision, Conceptualization.

## Funding

DPC was financially supported by 10.13039/501100001807São Paulo Research Foundation (10.13039/501100001807FAPESP) process numbers 2021/10434-4; 2022/03642-2. This work was supported by 10.13039/501100001807São Paulo Research Foundation (10.13039/501100001807FAPESP) process number 2018/25682-0.

LWB received a research fellowship from the São Paulo Research Foundation (10.13039/501100001807FAPESP) (grant 2025/00691-0).

Funding for DGO, 10.13039/100022797LRL, and genome sequencing was provided by the 10.13039/100014456Center for Bioenergy Innovation, supported by the U.S. Department of Energy, Office of Science, Biological and Environmental Research under Contract Number ERKP886.

## Declaration of competing interest

The authors declare that they have no known competing financial interests or personal relationships that could have appeared to influence the work reported in this paper.

## Data Availability

Research Link Provided. NCBIRe-sequencing of Clostridium thermocellum engineered strains (Original data)

## Footnotes

1 We use the following formatting conventions to distinguish between genes, proteins, enzymes and enzymatic reactions: Genes are shown in lower case italics (*gene*). Proteins and enzymes are shown in title case roman (Protein, Enzyme), and enzymatic reactions are shown in all caps (REACTION).
